# The prioritation and gap of preoperative COVID-19 vaccination in cancer surgery of the breast, head and neck, and skin: A cohort study of 367 patients in an Indonesian hospital

**DOI:** 10.1016/j.amsu.2021.103089

**Published:** 2021-11-18

**Authors:** Sumadi Lukman Anwar, Roby Cahyono, Herjuna Hardiyanto, Suwardjo Suwardjo, Darwito Darwito, Wirsma Arif Harahap

**Affiliations:** a Division of Surgical Oncology - Department of Surgery, Dr Sardjito Hospital / Faculty of Medicine, Public Health, and Nursing, Universitas Gadjah Mada, Yogyakarta, 55281, Indonesia; b Department of Surgery, Rumah Sakit Akademik (RSA), Universitas Gadjah Mada, Yogyakarta, 55291, Indonesia; c Division of Surgical Oncology - Department of Surgery, Dr M Jamil Hospital / Faculty of Medicine, Universitas Andalas, Padang, 25163, Indonesia

**Keywords:** Cancer patients, Surgery, COVID-19 vaccination, Postoperative complication

## Abstract

**Background:**

Postoperative infection of Coronavirus Disease 2019 (COVID-19) has been associated with higher risks of mortality and pulmonary complication. Preoperative vaccination could significantly prevent postoperative-related mortality and morbidity particularly for cancer patients.

**Methods:**

Cancer patients who were scheduled for elective major surgery were questioned for status and their willingness to receive COVID-19 vaccination and were prospectively monitored for the presence of postoperative COVID-19 infection and major complications.

**Results:**

During the period of April–July 2021, 367 patients with median age of 49 years were scheduled for cancer surgery. Procedures for breast cancer were the most frequently performed (N = 166, 45.2%). Surgery procedures with potential aerosol generating procedures (AGPs) were performed in total of 104 patients (28.3%). Only 6 of 367 patients (1.6%) were fully vaccinated in the day of surgery and 351 patients (95.6%) were willing to receive COVID-19 vaccination. Fully vaccinated patients were significantly higher among those who were living in urban areas (OR = 22.897, 95%CI:4.022–130.357, *P* = 0.0001). Willingness to get the COVID-19 vaccination was significantly higher among female patients (OR = 4.661, 95%CI:1.685–12.896, P = 0.003). Postoperative COVID-19 infection was confirmed in 17 patients (4.6%) and major surgical complications were observed in 12 patients (3.3%). None of preoperatively vaccinated patients experienced postoperative COVID-19 infection or the related major complications.

**Conclusion:**

Although prioritizing COVID-19 vaccination in preoperative cancer patients has been recommended to prevent postoperative fatalities, only a small proportion of our patients have been vaccinated. Preoperatively vaccinated patients show advantages in the prevention of postoperative COVID-19 infection and major surgery complications. The slow rollout and disparity in the vaccination progress for patients requiring a major cancer surgery need to be specifically addressed.

## Highlights

• Only small proportion of preoperative cancer patients have been fully vaccinated against COVID-19.
• There is rural-urban disparity in the preoperative COVID-19 vaccination of cancer patients.
• Certain comorbidities are associated with vaccine acceptance among cancer patients.
• Preoperative COVID-19 vaccination is potentially beneficial to prevent infection and complication.

## Abbreviations

AGP: Aerosol Generating Procedure
CI: Confidence Interval
COVID-19: Corona virus disease of 2019
HIV: Human Immunodeficiency Virus
HBV: Hepatitis-B Virus
HCV: Hepatitis-C Virus
OR: Odd Ratio
WHO: World Health Organization

## 1 Introduction

The prolonged COVID-19 pandemic has disrupted elective cancer surgery with frequent cancellation of non-essential procedures [[1], [2], [3]]. Technical assistance and recommendation for triage and prioritization of cancer surgery has been developed by several professional organizations to redirect hospital resources for COVID-19 management [[3], [4], [5]]. However, specific considerations related to deferring cancer surgery in developing countries need to compromise between prevention of ongoing COVID-19 transmission and preservation of patient's long-term prognosis [1]. Cancers in developing countries are often diagnosed in late stages [[6], [7], [8], [9]] and postponing surgery might have significant impact to the treatment outcomes and patients' quality of life. However, maintaining elective cancer surgery service during the pandemic will face several challenges. In the area with high levels of community transmission and elective surgery has been performed in COVID-19-free pathway, surgical patients are still at a high risk of COVID-19 infection [10] conferring them with significant higher rates of mortality and pulmonary complications after surgery [11,12].

The World Health Organization (WHO) has currently listed 12 vaccines for emergency use globally [13]. Implementation of preoperative vaccination has been associated with a reduced risk of postoperative pulmonary complications and mortality due to COVID-19 infection [11]. Relatively higher effectivity to save lives has been projected if the vaccination is prioritized to older cancer patients [11]. Because of the shortage in the availability of vaccines globally, low- and middle-income countries including Indonesia have to prioritize the populations at greater risk to receive the vaccine [11,14]. It is predicted that far-reaching coverage of the COVID-19 vaccination in developing countries will be achieved only until late 2022 [15].

Although the benefits of preoperative COVID-19 vaccination have been studied, the prioritization and rolling out for surgery patients are not yet manifested in some countries. In Indonesia, a national program for vaccination was initiated in January 2021 with prioritization for health care workers, the elderly, and public service workers [16]. After India experienced the second wave of COVID-19 pandemic with surging cases and mortality in March–June 2021 [17], Indonesia has also been affected by the second pandemic wave in the following months (June–July 2021) with daily cases over 50,000 including during the period of this study [18]. In the presence of high levels of community transmission, infection control and associated medical complications during postoperative care need to be well managed [11]. To maintain and re-establish elective cancer surgery services during and after the pandemic, preoperative vaccination is a very important step to support safe surgical care [11,19]. This study surveyed and estimated the status and gap in the preoperative COVID-19 vaccination of cancer patients within a hospital setting in a developing country.

## 2 Materials and methods

### 2.1 Study design

The study was performed by direct survey to preoperative cancer patients in an oncology clinic 1–7 days before the scheduled surgery. The planned procedures were performed in COVID-19-free surgical pathway in a referral hospital in Indonesia between April 1, 2021 and July 31, 2021. Presence of COVID-19 infection and postoperative complications were recorded during the period of the 30-days follow-up after surgery. The study protocol has been reviewed and approved by the institutional medical and health research ethics committee (Reference Number KE/FK/1129/2020) and has been reported in accordance with the STROCSS 2019 Guideline [20].

### 2.2 Patients

We surveyed all cancer patients scheduled for elective surgery in a hospital with COVID-free environment for surgical pathway. Only patients with planned major surgery during the time frame period were included. The hospital provided preoperative COVID-19 screening and swab testing for all cancer surgery patients and only those with negative results could proceed for elective surgery. Informed consent was collected from each patient. The baseline demographic data, diagnosis, and type of surgery were recorded from the electronic medical records. Fully vaccinated status was defined as receiving 2 doses of WHO-approved COVID-19 vaccines [13]. Patients were asked at the period of 1–7 days before the surgery for two questions:

- Have you been fully vaccinated with any type of WHO-approved COVID-19 vaccine? Yes/No
- Will you be willing to receive the COVID-19 vaccine? Yes/No

### 2.3 Follow-up

All patients were followed-up for a 30-day period after surgery for the presence of COVID-19 infection (using hospital checklist of COVID-19 screening, history taking, physical examination, and testing following the local procedure of COVID-19 assessment and testing pathway). We used Clavien-Dindo grade III-V as a measure of postoperative major complications. The secondary outcome measures were post-operative major complications including mortality, pulmonary complication, prolonged intensive care, unexpected ventilation, re-admission, and pneumonia during the 30-day period after surgery.

### 2.4 Statistical analysis

Survey responses were tabulated and then analyzed using SPSS 17.0 software (SPSS Inc., Chicago). Simple descriptive and frequency analyses were performed and are summarized in Table 1, Table 2, Table 3. Association of comorbidity with vaccination status and acceptance were analyzed using logistic regression.

**Table 1:** Baseline demographic data of cancer patients at the day of surgery and vaccination status (N = 367).

Variable	Category	N (percentage share)
Age	<15 years	2 (0.05%)
	15–35 years	57 (15.5%)
	35–55 years	194 (52.7%)
	55–75 years	104 (28.3%)
	>75 years	10 (2.7%)
Sex	Male	70 (19.1%
	Female	297 (80.9%)
Residence	Rural	334 (91.0%)
	Urban	33 (9.0%)
Location of cancer	Breast	166 (45.2%)
	Thyroid	67 (18.3%)
	Lymph node (biopsy and dissection)	35 (9.5%)
	Soft tissue sarcoma	32 (8.7%)
	Head and neck	26 (7.1%)
	Skin	24 (6.5%)
	Salivary gland	17 (4.6%)
Procedures	Wide excision (with/without skin flap or graft)	115 (31.4%)
	Mastectomy (with/without reconstruction)	103 (28.1%)
	Thyroidectomy	63 (17.2%)
	Lymphadenectomy	27 (7.4%)
	Modified radical neck dissection (mRND)	13 (3.5%)
	Parotidectomy	11 (2.7%)
	Lymph node dissection	11 (3%)
	Hemimandibulectomy	10 (2.7%)
	Limb amputation	7 (1.9%)
	Hemi-glossectomy and mRND	6 (1.6%)
	Hemimaxilectomy	1 (0.3%)
Comorbidity	Hypertension	81 (22.1%)
	Diabetes mellitus	49 (13.4%)
	Chronic infection	8 (2.2%)
	Autoimmune disease	2 (0.5%)
	Chronic renal disease	3 (0.8%)
	Cardiovascular disease	3 (0.8%)
	Dyslipidemia	10 (2.7%)
	BMI >25	108 (29.4%)
	Any comorbidity	122 (33.2%)
	Multiple comorbidities	29 (7.9%)
Vaccination status	Vaccinated	6 (1.6%)
	Not vaccinated	361 (98.4%)
Acceptance of COVID-19 vaccination	Yes	351 (95.6%)
	No	16 (4.4%)
Postoperative COVID infection	Yes	17 (4.6%)
	No	350 (95.4%)
Postoperative major complication	Yes	12 (3.3%)
	No	355 (96.7%)

**Table 2:** Odd ratios and 95% confidence intervals of vaccination status (complete vaccination in 6 patients, 1.6%) and the acceptance (willingness to receive vaccine in 351 patients, 95.6%) according to sex, residence, and older age.

Variable	Category	Vaccination status				Vaccination acceptance			
		Vaccinated	Not vaccinated	OR (95%CI)	*P* value	Yes	No	OR (95%CI)	*P* value
Sex	Female	5	292	1.182 (0.136–10.27)	0.880	289	8	4.661 (1.685–12.89)	**0.003**
	Male	1	69	ref		62	8	ref	
Residence	Urban	4	29	22.90 (4.022–130.4)	**0.0001**	30	3	0.405 (0.109–1.501)	0.176
	Rural	2	332	ref		321	13	ref	
Age	>60 y	2	67	2.194 (0.394–12.228)	0.370	59	10	0.121 (0.042–0.346)	**0.0001**
	≤60 y	4	294	ref		6	292	ref

**Table 3:** Association of vaccination status (complete vaccination in 6 patients, 1.6%) and the acceptance to receive COVID-19 vaccination (willingness in 351 patients, 95.6%) with medical comorbidity (-ies).

Variable	Category	Reference	Vaccination status		Acceptance to vaccination	
			OR (95%CI)	*P* Value	OR (95%CI)	*P* Value
Hypertension	Yes	No	0.702(0.081–6.100)	0.749	0.263(0.095–0.723)	**0.010**
Diabetes mellitus	Yes	No	1.304(0.149–11.40)	0.288	0.315(0.105–0.950)	**0.040**
Chronic infection	Yes	No	0	-	0	-
Autoimmune disease	Yes	No	0	-	0	-
Chronic renal disease	Yes	No	0	-	0.086(0.007–1.002)	**0.050**
Cardiovascular disease	Yes	No	0	-	0.086(0.007–1.002)	**0.050**
Dyslipidemia	Yes	No	0	-	0.395(0.047–3.321	0.392
BMI >25	Yes	No	2.438(0.484–8.430)	0.280	1.263(0.398–4.007)	0.692
Any comorbidity	Yes	No	0.397(0.046–3.343)	0.401	0.281(0.100–0.793)	**0.016**
Multiple comorbidities	Yes	No	2.379(0.269–21.07)	0.436	0.161(0.052–0.503)	**0.002**

## 3 Results

### 3.1 Patient's characteristics and surgery procedures

We were able to recruit 367 cancer patients scheduled for surgery during the period of 1 April 2021 to 31 July 2021. The majority of patients were female (N = 297, 80.9%) and surgery procedures in the breast were the most frequently performed (N = 166, 45.3%, Table 1). Surgery in the thyroid, lymph nodes, soft tissue sarcoma, head and neck, skin, and salivary glands were performed in 18.3%, 9.5%, 8.7%, 7.1%, 6.5%, 6.5%, and 4.6%, respectively. Wide excision with/or without skin flap or skin graft was the most common surgery procedures during the study (N = 115, N = 31.4%). Mastectomy and thyroidectomy accounted for almost half of the surgical procedures in this study (28.1% and 17.2%, respectively). Aerosol generating procedures (AGPs) during the process of general anesthesia and surgery were potentially found in the procedures of thyroidectomy, neck dissection, parotidectomy, hemimandibulectomy, hemoglossectomy, and hemimaxilectomy (in a total of 104 patients, 28.3%).

Safety and efficacy of COVID-19 vaccination vary among populations with complex medical comorbidities. Among preoperative cancer patients in this study (N = 367), 22.1% (N = 81) had hypertension (Grade 1–3), 13.4% (N = 49) had type-2 diabetes, 2.2% (N = 8) had chronic infection (human immunodeficiency virus infection, hepatitis-B infection, and tuberculosis), 0.5% (N = 2) had autoimmune diseases (Sjogren's syndrome and systemic lupus erythematosus), 0.8% (N = 3) had chronic renal diseases (hydronephrosis), 0.8% (N = 3) had cardiovascular diseases (stroke, congestive heart failure), 2.7% (N = 10) had dyslipidemia, and 29.4% (N = 108) patients were overweight or obese. In total, 122 (33.2%) patients had at least one medical comorbidity and 29 (7.9%) patients had multiple comorbidities (Table 1).

### 3.2 Status and acceptance of COVID-19 vaccination

Among 367 perioperative cancer patients, only 6 (1.6%) were fully vaccinated against COVID-19. In the first face of COVID-19 vaccination program in Indonesia, inactivated virus vaccines were first deployed. All preoperative fully vaccinated patients (N = 6) received Sinovac vaccines. We found no significant different between male and female patients in the status of preoperative vaccination (*P* = 0.880, Table 2). Patients who lived in urban areas were significantly associated with higher chance to be fully vaccinated before cancer surgery compared to those who lived in rural areas (OR = 22.897, 95%CI:4.022–130.357, *P* = 0.0001). Of all patients, 95.6% (N = 351) expressed that they were willing to take COVID-19 vaccine. Acceptance to COVID-19 vaccination was significantly higher among female patients (OR = 4.661, 95%CI = 1.685–12.896, *P* = 0.003, Table 2). Vaccination acceptance was not associated with patients’ residence in rural and urban areas.

### 3.3 Association of status and acceptance of COVID-19 vaccination with medical comorbidities

Because of the high degree of hesitancy to get the COVID-19 vaccination due to safety concerns in populations with certain degrees of medical comorbidities, we analyzed their associations with status and willingness to get the vaccination among preoperative cancer patients. No significant association of vaccination status with the presence of hypertension, diabetes mellitus, and overweight (Table 3). In our study, all patients with chronic infection, autoimmune diseases, chronic renal disease, cardiovascular diseases, and dyslipidemia were not yet vaccinated (Table 3). The presence of any comorbidity and multiple comorbidities were also not significantly associated with complete vaccination status. Willingness to receive COVID-19 vaccine was significantly lower in cancer patients with the following comorbidities: hypertension (OR = 0.263, 95%CI:0.095–0.723, *P* = 0.010), diabetes mellitus (OR = 0.315, 95%CI:0.105–0.950, *P* = 0.040), chronic renal diseases (OR = 0.086, 95%CI:0.007–1.002, *P* = 0.050), cardiovascular diseases (OR = 0.086, 95%CI:0.007–1.002, *P* = 0.050). Cancer patients with any comorbidity also had lower willingness to get the COVID-19 vaccination (OR = 0.281, 95%CI:0.100–0.793, *P* = 0.016), and the acceptance was much lower in the presence of multiple comorbidities (OR = 0.161, 95%CI:0.052–0.503, *P* = 0.002, Table 3).

### 3.4 Association of status and acceptance of vaccination with postoperative COVID-19 infection and major complications

Among 367 cancer patients, 17 patients (4.6%) 30-day postoperative patients were confirmed with COVID-19 infection. Major medical complications were observed in 12 patients (3.3%), including 5 patients died due to COVID-19 infection (1.4%), 6 patients with pulmonary complications (1.6%), 1 patient with prolong postoperative intensive care (0.3%), and 1 patient with hospital readmission (0.3%) within 30-day after surgery. Mortality rate of postoperatively COVID-19 infected patients was 29% (5 of 17). In this study, no patient with preoperative fully vaccinated status experienced postoperative COVID-19 infection and major surgery complications. Using multivariable regression, we did not find significant association preexistence of comorbidities among non-vaccinated patients for postoperative COVID-19 infection and major surgery complications.

## 4 Discussion

One in a thousand of the COVID-19-related mortalities could be prevented with preoperative vaccination [11]. The number of lives that could be saved with preoperative vaccination could be even higher in older cancer patients [11]. This study surveyed status and willingness of getting the COVID-19 vaccination among preoperative cancer patients and prospectively observed the presence of COVID-19 infection and major surgery complications within period of 30-day after surgery. In our study, half patients were at age of 35–55 years (Table 1) and 4.6% (N = 17) were older than 70 years that were most benefited from vaccination to prevent COVID-19 related mortality [11]. Surgery procedures with potential AGPs were performed in 28.2% patients causing higher risks of COVID-19 transmission to healthcare workers and other surgery patients in the ward. Although elective surgery was performed in COVID-19-free pathway, there was still a risk of cross-infection from patients in the incubation period who had tested negative at admission [21] and the risk was even higher in procedures involving AGPs.

Cancer has been considered as a comorbidity for COVID-19 patients with three times higher risk of death [1,10]. Pre-existing comorbidities in cancer patients have been associated with high risks of transmission and poorer outcome after contracting COVID-19 [21]. Hypertension and diabetes were the most common additional comorbidities in this study (22.1% and 13.4%, respectively). Additional pre-existing comorbidities in cancer patients will predispose them to higher medical complications after surgery in association with COVID-19 infection [10,21,22]. In addition to the elevated COVID-19 transmission [21,22], metabolic comorbidities per se have adverse association with long-term prognosis of cancer patients [23]. Surgery itself is also associated with higher risk of virus exposure due to proinflammatory cytokines and mechanical ventilation [22]. In patients with advanced cancer stages at diagnosis as commonly found in developing countries, systemic proinflammatory markers are usually also higher [24] that might increase risks of complication after surgery. Preexisting comorbidities have been associated with higher hesitancy to receive COVID-19 vaccination due to safety concerns [11,14]. In the beginning of the COVID-19 rollout, ageism emerged as an important issue in Indonesia since elderly were often positioned at the back of queue [25]. With high incidence of additional comorbidities (33.2%, N = 122, Table 1) among cancer patients, the queue and willingness to receive the COVID-19 vaccine might cause wider vaccination gap.

We identified a significant vaccination gap between preoperative cancer patients living in the rural and urban areas (OR 22.9, Table 2). Although a similar disparity has also been reported in high income countries [26], policy priority and equitable vaccine distribution need to be continuously improved in Indonesia. We also identified significantly lower acceptance of COVID-19 vaccine among males and cancer patients older than 60 years (Table 2). Harapan et al. also identified lower COVID-19 vaccine acceptance among males in general population in Southeast Asia regions although older age and residence in rural were not associated with the acceptance [14]. Several variables and matrices related to vaccine acceptance and hesitancy have been reported globally [27] with certain various degrees among countries. Responses to the pandemic including universal vaccination program by a country and their citizen acceptance are also largely influenced by social and economic factors [28,29]. Concerns about vaccine efficacy in cancer patients, overwhelming media coverage of vaccine side effects, and vaccine policy and prioritization are among other factors influencing COVID-19 acceptance [14].

Although frequency of medical comorbidity among preoperative cancer patients was relatively high (Table 1), we did not find any significant association with status of vaccination completeness (Table 3). The proportion of fully vaccinated preoperative patients was very low in this study that might be caused both those with and without comorbidities who were not yet vaccinated. Comorbidity increases risks of infection, severely illness, and death, thus in the scenario of limited vaccine availability, populations with comorbidities should be prioritized [30]. Cancer itself is a comorbid condition that has 7 times increased risk to be infected [31] and usually with worse COVID-19-related outcomes. Therefore, cancer patients should be considered to be put in the first queue for COVID-19 vaccination [10,11]. Preexisting medical comorbidities in cancer patients including elderly, diabetes, overweight, and hypertension should also warrant the prioritization [11,30]. However, we identified lower willingness to get the COVID-19 vaccination in cancer patients with additional comorbidities of hypertension, chronic renal diseases, and cardiovascular diseases (Table 3). Cancer patients with multiple comorbidities also had significantly lower willingness to get the COVID-19 vaccine. Chan et al. [32] also reported lower intention for COVID-19 vaccination among cancer patients in Hong Kong because of critical concerns to the safety and efficacy. Higher hesitancy to COVID-19 vaccination was also reported among breast cancer patients in Mexico [33]. With the disparity of vaccination status and acceptance among cancer patients, devoted strategies are required to build vaccine literacy and health system readiness to deliver universal vaccination. To educate about the advantages of vaccination to patients undergoing cancer surgery, active participation of oncologists particularly cancer surgeons is very essential [33].

We observed that preoperative fully vaccinated patients did not experience COVID-19 infection and major surgery complications. It has been reported that 0.6–1.6% patients are infected with COVID-19 virus after undergoing elective surgery thus predisposing them with up to 8 times increased risk of mortality in the 30 days after surgery [11,22]. In this study, we confirmed 4.6% postoperative COVID-19 infection almost triple than previously reported [11]. The risks of perioperative COVID-19 infection could be higher during the peak of second pandemic wave as the escalating community transmission. This study was performed during the period of second wave COVID-19 pandemic in Indonesia (April–July 2021) with daily newly infected cases and mortality recorded to 50,000 and 1,5000, respectively [18]. In our study, postoperative COVID-19 infection caused 29.4% deaths (5 of 17 patients). A multicenter prospective study involving emergency and elective surgery procedures reported a mortality rate of 25.6% (205 of 800) of postoperatively infected patients [22]. Although only elective surgery patients were recruited in this study, we reported higher incidence of postoperative COVID-19 infection as well as higher mortality rate than previous reports [22,34]. Therefore, institutional and structural refinement is required to ensure a safe surgery framework during the COVID-19 pandemic including prioritization of preoperative vaccination.

This study provides a snapshot of a gap of COVID-19 vaccination status and acceptance among preoperative cancer patients in a developing country. Evaluation of postoperative COVID-19 infection and major surgery complications indicates the magnitude advantages of preoperative vaccination to further support safe surgery procedures. Some limitations of this study are associated with a single center study, number of study participants, and lower proportion of fully vaccinated patients. We used a period of 30 days after surgery to monitor postoperative infection and complication thus sources of virus transmission might come from hospital admission and community. This study had some limitations since only 6 patients (1.4%) patients were fully vaccinated before the surgery that prevented some statistical tests. In addition, we surveyed patients directly to assess acceptance or hesitancy to get the COVID-19 vaccination and did not provide further follow-up. A multicenter study with additional exploration in the intended acceptance or hesitance to vaccination will provide more accurate delineation of the advantages as well as challenges of preoperative vaccination to support reinitiating safe elective surgery procedures in the resilience period after COVID-19 pandemic.

## 5 Conclusions

Only very small proportion of preoperative cancer patients have been fully vaccinated indicating the gap in prioritization rolling-out vaccination system that should be immediately addressed. Our study also identifies demographic and social inequality in the vaccination status and acceptance among preoperative cancer patients. Although larger studies are required, we have shown the potential advantages of preoperative vaccination to prevent postoperative COVID-19 infection and major surgery complications.

## Provenance and peer review

Not commissioned, externally peer reviewed

## Sources of funding

SLA received Tahir Foundation seed grant (1/2020).

## Ethical Approval

The study protocol has been reviewed and approved by the institutional medical and health research ethics committee (Reference Number KE/FK/1129/2020).

## Author contribution

SLA conceptualized the first draft. SLA, RC collected and analyzed the data. SS, HH, DD, and WAH provided suggestions, feedback, and revised the manuscript. All authors read and approved the final draft.

## Registration of Research Studies

1. Name of the registry:Research Registry
2. Unique Identifying number or registration ID: reasearchregistry7116
3. Hyperlink to your specific registration (must be publicly accessible and will be checked): https://www.researchregistry.com/browse-the-registry#home/registrationdetails/61309f44289dfb001eebae4c/

## Guarantor

SLA (Sumadi Lukman Anwar).

## Declaration of competing interest

We declare that no potential conflict of interest exists
